# Gender inequity in the medical profession: the women doctors in Spain (WOMEDS) study

**DOI:** 10.1186/s12960-023-00860-2

**Published:** 2023-09-20

**Authors:** Claudia Santucci, Beatriz González López-Valcarcel, Cristina Avendaño-Solá, Mari Carmen Bautista, Carmen Gallardo Pino, Lourdes Lledó García, Elena Martín-Perez, Pilar Garrido López

**Affiliations:** 1 Department of Quantitative Methods for Economics and Management, University of Las Palmas, Las Palmas de Gran Canaria, Canary Islands, Spain; 2 Department of Clinical Sciences and Community Health, University of Milan, Via Giovanni Celoria 22, 20133 Milan, Italy; 3 Department of Clinical Pharmacology, Hospital Universitario Puerta de Hierro-Majadahonda, Madrid, Spain; 4 Medical Council of Las Palmas, Spain and University of Las Palmas de Gran Canaria, Las Palmas de Gran Canaria, Spain; 5 Faculty of Health Sciences, Rey Juan Carlos University, Alcorcón, Madrid, Spain; 6 Faculty of Medicine and Health Sciences, Alcalá University, Alcalá de Henares, Madrid, Spain; 7 Department of Surgery, Hospital Universitario la Princesa, Madrid, Spain; 8 Department of Medical Oncology, Hospital Universitario Ramón y Cajal, Madrid, Spain; 10 FACME (Federación Asociaciones Científico Médicas Españolas), Madrid, Spain

**Keywords:** Gender inequity, Health Services Research, Gender inequity in medicine, Feminization of medicine, Desigualdades de género, Investigación en Servicios de Salud, Sesgo de género, Feminización de la medicina

## Abstract

**Background:**

The long-standing underrepresentation of women in leadership positions in medicine is well-known, but poorly documented globally. There is some evidence of the gender gap in academia, medical society leadership, or specific problems in some specialties. However, there are no investigations analyzing all medical specialties together and reporting the glass ceiling from a 360º perspective that includes positions in academia, research, professional organizations, and clinical activity. Additionally, the majority of studies have a US perspective, and we wonder if the perspective of a European country might be different. The WOmen in MEDicine in Spain (WOMEDS) project (https://womeds.es) aims to describe and characterize, in a systematic and detailed way, the gender bias in the medical profession in Spain in order to monitor its evolution over time and contribute to prioritizing gender policies.

**Methods:**

We retrieved data for the calendar years 2019–2021 from several sources and selected surveys. We built four groups of indicators to describe leadership positions in the medical profession: (i) leadership in healthcare according to specialty and region; (ii) leadership in scientific and professional bodies; (iii) academic career; and (iv) leadership in clinical research activity. As a summary measure, we reported the women ratios, calculated as the percentage of women in specific top positions divided by the percentage of women in the relevant population.

**Results:**

We found gender inequity in leadership positions in all four settings. During the observed period, only 27.6% of the heads of departments in hospitals were women compared to 61.1% of women in medical staff. Ten of the 46 medical societies grouped in the Spanish Federation of Medical Societies (FACME) (21.7%) had a women president at some point during the study period, and only 4 annual congresses had ratios of women speakers higher than 1. Women were over-represented in the lower positions and underrepresented in the top academic ones. Only 26% and 27%, respectively, of the heads of departments and deans were women. The applications for public funding for research projects are led by women only in 45% of the cases, and the budget granted to women in public calls was 24.3% lower than that of men.

**Conclusion:**

In all the areas analyzed, the leadership positions are still mostly occupied by men despite the feminization of medicine in Spain. The severe gender inequity found calls for urgent interventions within a defined time horizon. Such measures must concern all levels, from national or regional regulation to changes in organizational culture or incentives in specific organizations.

**Supplementary Information:**

The online version contains supplementary material available at 10.1186/s12960-023-00860-2.

## RESUMEN EN ESPAÑOL

**Antecedentes:**

La prolongada infrarrepresentación de las mujeres en los puestos de liderazgo en medicina es bien conocida, pero está poco documentada de forma global. Hay evidencia sobre la brecha de género en la universidad, en el liderazgo en sociedades médicas o en determinadas especialidades. Sin embargo, no hay investigaciones que analicen el techo de cristal de cada una de las especialidades médicas desde una perspectiva 360º que incluya el liderazgo en la universidad, en la investigación con fondos públicos, en la representación en sociedades científicas y colegios profesionales y en la actividad clínica. Además, la mayoría de los estudios tienen una perspectiva estadounidense y nos preguntamos si la perspectiva de un país europeo podría ser diferente. El proyecto Mujeres en Medicina en España (WOMEDS) (https://womeds.es) tiene como objetivo describir y caracterizar de forma sistemática y detallada sesgo de género en la profesión médica en España, para monitorizar su evolución en el tiempo y contribuir a priorizar las políticas de género.

**Métodos:**

Construimos cuatro grupos de indicadores sobre liderazgo de mujeres médicos: (i) en la asistencia sanitaria; (ii) en las organizaciones científicas y profesionales; (iii) carrera académica, y; and (iv) l en la investigación basándonos en datos públicos y resultados de encuestas propias s referidas a los años 2019–2021. Como medida de análisis, calculamos los ratios de mujeres, definidos como el porcentaje de mujeres en puestos altos específicos dividido por el porcentaje de mujeres en la población relevante.

**Resultados:**

Encontramos un sesgo de género en los cuatro ámbitos. Durante el periodo observado, solo el 27.6% de los jefes de servicio de los hospitales eran mujeres, frente al 61.1% de mujeres en la plantilla. Diez de las 46 sociedades médicas agrupadas en la Federación de Asociaciones Científico Médicas Españolas (FACME) (21.7%) tuvieron una mujer como presidente en algún momento del periodo de estudio y sólo 4 congresos anuales tenían ratios de mujeres ponentes superiores a 1. Las mujeres estaban sobrerepresentadas en los cargos inferiores e infrarrepresentadas en los cargos académicos superiores. Sólo el 26% y el 27%, respectivamente, de los jefes de departamento y decanos eran mujeres. La solicitud de proyectos de investigación con financiación pública fue liderada por mujeres en un 45% de los casos y la financiación media de los proyectos concedidos a las mujeres fue un 24.3% inferior a la de los hombres.

**Conclusión:**

En todos los ámbitos analizados, las posiciones de liderazgo siguen siendo mayoritariamente ocupada por varones a pesar de la feminización de la medicina. Para cambiar esto, será necesario tomar medidas, tanto regulatorias -a nivel nacional y nacional regional como promover cambios en la cultura organizativa o en los incentivos en organizaciones concretas.

## Background

Over the last few decades, there has been a steady increase in the number and percentage of women in medicine, resulting in a more feminized profession. However, female representation in decision-making positions remains low on a global scale [1, 2].

In academic careers, women are internationally underrepresented, especially in senior positions [3–5]. Similarly, in many the medical societies across variuos specialties, gender inequity persists, not in terms of membership but in terms of recognition and appointment to leadership positions [6]. Additionally, in academic medicine, including research and teaching, women are underrepresented on an international scale. In the United States, women are less likely to be promoted to associate professor, full professor, and department chair, and this bias has persisted, with the gap widening in recent years for full professorship [7, 8]. These indicators highlight the existing gender inequity in medical professional societies. Other indicators include the underrepresentation of women in medical board membership, relevant roles in congress programs, and prominent positions in teaching and publications. Discrimination is not always explicit, there is evidence of differing language used based on gender within the context of international medical conferences. For example, women tend to introduce speakers using their professional title, regardless of gender, while men introduce female speakers with professional titles less frequently than they do male speakers [9, 10]. Female physicians continue to face numerous challenges in medicine, ranging from implicit bias to barriers in promotion, responsibility, and payment gaps. Consequently, despite an equal number of men and women graduating from medical schools, only a small fraction of female physicians ascend to medical leadership positions [11, 12].

There is gender inequity in the academic publishing system, with a systematic underrepresentation of women as authors, referees, and editors [13–16]. Gender bias in scientific publications and its causes or mechanisms has been studied in different fields. The composition of editorial committees and the pool of referees by sex could make a difference. It has also been investigated whether the editorial processes, which are a set of interlinked decisions, may have any direct or indirect effect on the lower rate of publications by women. A recent study examining gender bias in 145 peer-reviewed scientific journals surprisingly found that manuscripts written by women received systematically more positive reviews, and that manuscripts with a higher proportion of women among authors were accepted more frequently, although there are some differences between fields of research [17]. Moreover, the study found that women are systematically less involved in peer review and are rarely appointed to prestigious editorial positions. In December 2017, the Lancet group launched the #LancetWomen project, focusing on the roles of women in editing, reviewing, and authoring articles. Following its findings, some of the group’s journals have expanded their editorial boards to include more women [18].

Although the international evidence is extensive, gender bias in medicine has not been properly studied in Spain. Local studies focused on specific medical specialties have pointed to a pronounced inequity both in healthcare practice and in academic medicine [15, 19–25]. In the 2015–2016 academic year, 65.7% of undergraduate medical students in Spain were women, and by 2020–2021, that percentage increased to 69.4% [26]. Currently, more than half of the physicians in Spain are women (around 58%), and this trend is increasing [12, 27]. This percentage is higher among medical doctors aged 35 or younger (67%), and varies among regions, ranging from 341/100,000 population in Melilla to 575 in Aragon. In 2021, over 60% of physicians working in the public network of hospitals and health centers were women, with variability related to different specialties [28]. Official figures for the number of physicians working in private practice in Spain are not yet available.

To gain further insights into the current representation of women in leadership roles and to extend our understanding of gender challenges in Spain, the Women in MEDicine in Spain (WOMEDS) project was initiated. The primary goal of this project is to systematically and comprehensively describe and characterize gender bias in the medical profession in Spain, in order to monitor its evolution over time and contribute to prioritizing gender policies. The project focuses on the clinical setting but also has implications for professional organizations, academia, and research. Four groups of indicators were established: (i) healthcare; (ii) scientific and professional bodies (medical councils, medical associations, and medical conferences); (iii) academic career (universities); and (iv) research career, concerning women physicians for each specialty.

## Methods

### Data sources and variables

The project was proposed by the *Federación de Asociaciones Científico Médicas Españolas* (FACME), a non-profit organization representing 46 Spanish medical societies corresponding to the different medical specialties. Once the project was approved, a core multidisciplinary team was defined, establishing the items to be collected and the specific sources that were available, including unpublished data. Table 1 contains the list of collected variables classified by groups. We retrieved data from various sources, including the medical societies included in FACME, universities, Medical Councils, National Public Research Institute (*Instituto de Salud Carlos III* (ISCIII)), and the Regional Health Systems that operate and manage the public National Health System in Spain. Healthcare information is disaggregated by medical specialties in most regions.

**Table 1:** List of primary variables collected, by group of indicators

Group	Primary data collected	Years	Disaggregation	Source
1. Healthcare	• Total number of physicians (staff) in healthcare in the public network (men) • Total number of physicians (staff) in healthcare in the public network (women) • Number of chiefs of department (men) • Number of chiefs of department (women) • Number of chiefs of section (men) • Number of chiefs of section (women)	2019–21	7 regions provided data by medical specialties (Aragón, Castilla Leon, Extremadura, Galicia, Madrid, Murcia, Navarra)	Regional governments (departments of health or regional health services)
2. Medical councils, associations and conferences	• Number of members of the society (men) aged under 50 • Number of members of the society (men) aged over 50 • Number of members of the society (women) aged under 50 • Number of members of the society (women) aged over 50 • Sex of the president (man/woman) • Number of members of the board (men) • Number of members of the board (women) • Sex of the scientific coordinator of the annual conference • Number of members of the scientific committee, annual conference (men) • Number of members of the scientific committee, annual conference (women) • Number of invited speakers to the annual conference (men) • Number of invited speakers to the annual conference (women)	2019–21	By scientific societies (47)	FACME
	• Number of members of the board of directors (men) • Number of members of the board of directors (women) • Sex of the president • Sex of the secretary • Sex of vice-presidents • Sex of vice-secretary • Sex of the treasurer	2021	By provinces (50)	Medical councils
	• Total number of full, honorary, corresponding, other members (men) • Total number of full, honorary, corresponding, other members (women) • Number of members of the board of directors (men) • Number of members of the board of directors (women) • Sex of the president • Sex of the secretary	2021	By regional academies (12)	Academies of Medicine
	• Number of members of the board of directors (men) • Number of members of the board of directors (women) • Sex of the president • Sex of the secretary	2021	By regions (17) plus the State Confederation	Medical Unions
3. Academic career in universities	• Number of teaching staff by categories (men) • Number of teaching staff by categories (women) • Number of chiefs of department (men) • Number of chiefs of department (women) • Number of deans (men) • Number of deans (women) • Number of advisors of doctoral thesis 2020–21 (men) • Number of PhD thesis supervisors in 2020–21 (men) • Number of PhD thesis supervisors in 2020–21 (women)	2021–22	Aggregated data for 39 out of the 44 medical schools	Universities (Conference of Deans of medical schools)
4. Research	• Number of applications headed by men • Number of applications headed by women • Number of granted projects headed by men • Number of granted projects headed by women • Average funding per project headed by a man • Average funding per project headed by a woman	2019–20	By specific project call belonging to the State Subprogram for Knowledge Generation^a^	Instituto de Salud Carlos III

^a^Technological Development Projects in Health; Health Research Projects; International Joint Programming; Independent Clinical Research Projects

Regarding health care activity (group 1), we gathered primary data on the composition of medical departments and the percentage of women to men in leadership positions, by medical specialty and Regional Health System (Autonomous Communities). For group 2, FACME sent out a specific survey to collect information focused on the medical societies, such as the gender membership ratio, the percentage of women as invited speakers in congresses, presidents, and board members for the calendar years 2019–2021. Details on Councils and medical academies were obtained from public sources. A specific survey was also designed and distributed to the medical schools to gather information on the teaching staff by gender and category, as well as the proportion of women holding the position of head of department and dean in the different Spanish universities for the academic medical courses related to 2020–2021 (group 3). For the research (group 4), ISCIII provided gender information about human resources and health research projects applied for and awarded in national public competitive calls in 2019–2020 and funding.

### The indicators

From the original variables we defined, when relevant, the women ratio (WR), taking into account the variability of the proportion of women, which is strictly related to the medical specialty. In fact, medical specialties are heterogeneously feminized in Spain [28]. For example, in 2021, only 28.5% of urologists were women, while over 75% of pediatricians and obstetricians/gynecologists were women. Thus, the WR is calculated as follows:

$$WR=\frac{\mathrm{\%\, Women\, in\, the\, specific\, position\, under\, analysis}}{\mathrm{\%\, Women\, in\, the\, relevant\, population}},$$

The WR proxies gender imbalances in power and influential positions in healthcare, medical associations and organizations, universities, and research programs. When relevant and possible, it was calculated according to regions and medical specialties. A WR equal to 1 identifies a gender balance in the specific position under analysis, as the proportion of women in that position equals the proportion of women in the relevant population. A WR below 1 identifies an inequity against women, while a WR above the unity indicates an inequity in favor of women.

For the first group of indicators, we computed the percentage of women working in public healthcare centers and the WR for head of department and section overall in Spain, as well as across Spanish regions: $\frac{\mathrm{\%\, Head\, women}}{\mathrm{\%\, Women\, in\, the\, speciality}}$, when available, WR was disaggregated according to medical specialties.

Regarding the second group of indicators, we reported the WR for speakers in national medical congresses, the WR for members in the scientific committees, the percentage of women on the board of directors, and the percentage of female presidents for each national medical society in Spain involved in FACME. For the official colleges of physicians, we reported the percentage of women presidents. The feminization in academies of medicine across regions was also considered.

The indicators for academic positions included the WR for full and associate professors in medical schools, as well as the percentage of women in relevant positions such as deans of medical schools and head of departments.

For the fourth set of indicators, we analyzed gender information about different publicly funded Research Programs from the perspective of Human Resources and Research Projects. We used data from the Center of Biomedical Research Network, the Platforms for Research Support, and different calls for Health Research Projects. We also reported the budget granted in the different State Knowledge Generation Subprogram projects, including Technological Development Projects in Health, Health Research Projects, Independent Clinical Research Projects, and AC International Joint Programming. We calculated the percentage of projects headed by a woman, both those admitted and accepted, along with the corresponding WR. Additionally, we examined the difference in funding allocated to women and men in the different calls.

## Results

Out of 17 Autonomous Communities (AACC) of Spain, we received the requested information from 14 of them, representing 71% of the population of Spain in 2021. Among these AACCs, 7 provided us with disaggregated data by specialties. We received responses from 38 medical associations affiliated with FACME. We did not encounter any missing data from the 50 medical councils and academies of medicine. Regarding universities, we collected data from 39 out of the 44 medical schools in Spain.

### First group of indicators: healthcare

**Fig. 1:**
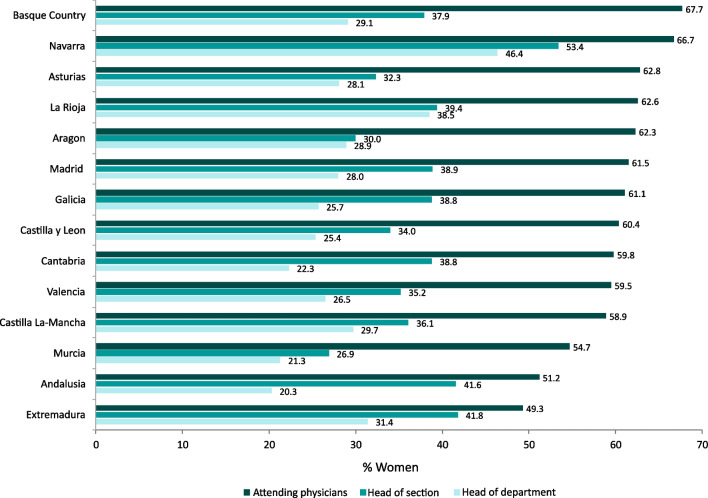
Percentage of women attending physicians, head of department, and head of section according to Spanish regions, 2019–2021

Figure 1 displays the global percentage of women medical doctors, women heads of departments, and women heads of section/unit (referred to as “section” hereafter) in Spanish regions (AACC) during 2019–2021. The overall female percentage of attending physicians for the 14 AACC regions included in our study was 61.1%. It ranged from 49.3% in Extremadura to 67.7% in the Basque Country. The percentages of women in leadership positions as heads of department and section were lower than those of women in the medical workforce across all regions. The percentage of women heads of department varied from 20.3% in Andalusia to 46.4% in Navarra, while the percentage of women heads of sections ranged from 26.9% in Murcia to 53.4% in Navarra.

A detailed analysis of women empowerment in heads of department and section across seven AACC regions, disaggregated by specialty, revealed great variability, with no clear patterns observed across specialties regarding gender distribution among heads (data not shown). Furthermore, there was no clear distinction between medical and surgical specialties, despite most surgical specialties exhibiting low level of female representation. In most of the considered specialties within the seven regions, the representation of women was lower than the unit, except for Preventive Medicine. This specialty showed a favorable representation of women in five out of the seven AACCs analyzed (data not shown).

### Second group of indicators: scientific and professional bodies

**Fig. 2:**
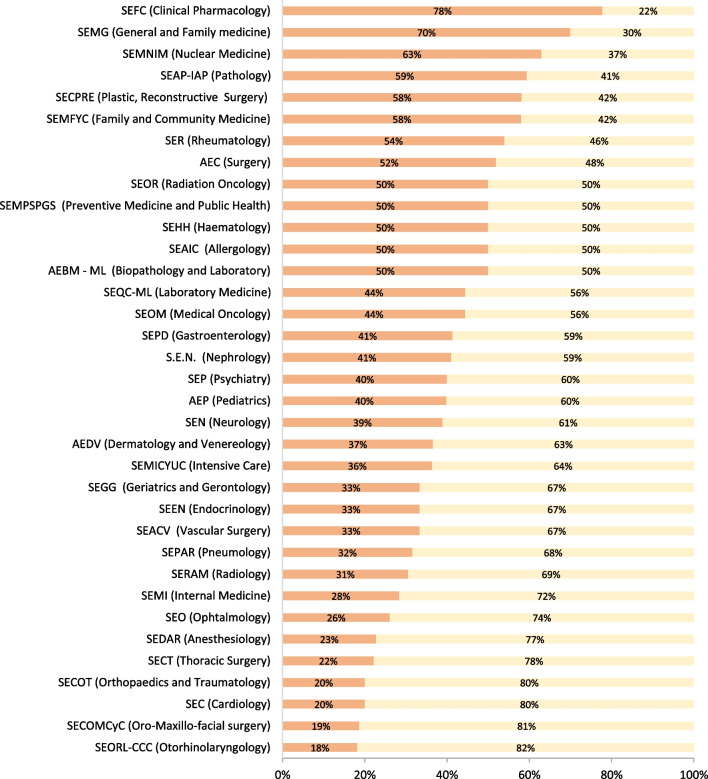
Percentage of women (orange) and men (yellow) in the Executive Board of Scientific Societies during 2019–2021

During the period 2019–2021, the percentage of women in the board of director across Spanish medical society varied between 18% in otorhinolaryngology and 78% in clinical pharmacology (Fig. 2). Additional file 1: Fig. S1 reveals the WRs for speakers in national medical congresses in 2019–2021 organized by the national medical associations ranged from 0.21 to 1.21. Among the 22 societies included, four societies reported a WR higher than 1, namely SECT (Spanish Society for Thoracic Surgery), SEMNM (Spanish Society of Nuclear Medicine), SEC (Spanish Society of Cardiology), and AEDV (Spanish Society of Dermatology and Venereology). Additional file 1: Fig. S2 provides information on the WR for members in the scientific committees of national congresses during the same period. The WR values ranged from 0.35 to 1.34. Only five medical societies exhibited a WR greater than one: SEAP-IAP (Spanish Society of Pathology), SEMNM, SEMG (Spanish Society for General and Family Physicians), SECPRE (Spanish Society of Plastic, Reconstructive and Aesthetic Surgery), and SEAIC (Spanish Society of Allergology). In terms of the percentage of women presidents of the medical societies, Additional file 1: Table S1 reveals that throughout the entire period, only three medical societies—SEFC (Spanish Society of Clinical Pharmacology), SEN (Spanish Society of Nephrology), and SEQC-ML (Spanish Society of Laboratory Medicine)—had a woman serving as president. Additional file 1: Fig. S3 presents the percentages of women on the board of directors among the official colleges of physicians by provinces. The data displayed in this figure demonstrate that the percentage of women varied between 11% in Burgos and 64.3% in Cantabria in 2021. Similarly, Additional file 1: Fig. S4 provides information on the percentage of women on board of directors in academies of medicine, where the women percentage varied from 0% in Salamanca, Sevilla, Asturias, Galicia, and Valladolid to 50% in Catalonia.

### Third group of indicators: academic positions in universities

**Fig. 3:**
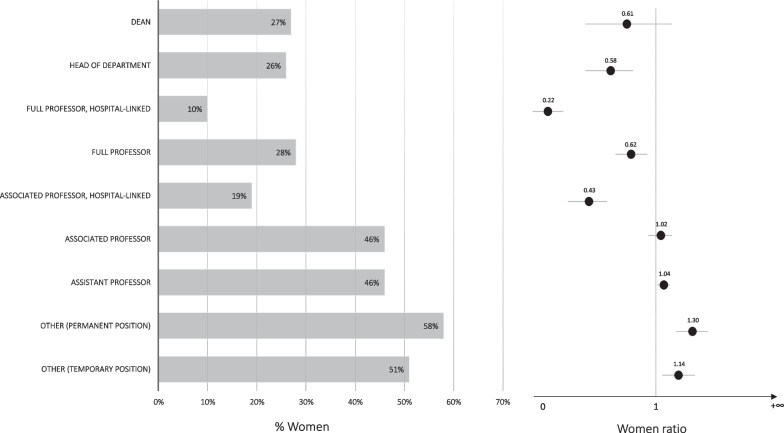
Percentage of women holding academic positions in Spanish universities of medicine and the corresponding women ratio, 2020–2021

Figure 3 illustrates the distribution of academic positions in medicine universities based on gender, along with the corresponding WRs. Concerning permanent positions, the 10% of the tenured university professors in the top position with a salary supplement for clinical activity were women, resulting in a WR for full professor, hospital-linked, of 0.22. On the other hand, 58% of the lowest positions were held by women, leading to a WR for other temporary positions of 1.14. The percentages of women head of department and dean in the academic year 2020–2021 were 26% and 27%, respectively.

### Fourth group of indicators: research

In 2020, fewer women physician applied to the State Subprogram for Knowledge Generation ISCIII’s calls as compared to men. Among all the calls, only 45% were led by women, with the lowest percentage recorded in the independent clinical research modality, where only 22% of the applications were headed by physician women (Table 2 and Additional file 1: Figs. S5–S9). The granting rate for women was lower overall (38% vs. 41%) and in each of the calls. Additionally, the average funding for projects awarded to physician women was 24.3% lower than that of men, except for the international joint programming modality, where women received 11% more budget than men. In the Technological development projects, AC International Joint Programming, and Independent clinical research projects, the percentage of admission and granted projects was two times higher for men than for women, while the difference in the success rate was less evident but still in favor of men.

**Table 2:** Total number of projects submitted and granted along with the average public funding according to sex, 2020

	Projects submitted		Projects granted		Average funding per project	
	Total *n*	Women *%*	Total *n*	Women *%*	Women *€*	Men *€*
State knowledge generation subprogram overall data	1825	45.4	722	44.0	119,784	158,212
Technological development projects in health	112	35.7	25	32.0	83,126	90,305
Health research projects	1501	48.6	632	46.7	113,479	127,026
AC International joint programming	106	34.9	31	29.0	159,686	143,680
Independent clinical research projects	106	21.7	34	17.6	418,804	586,195

Table 3 provides information on the total number of medical doctors and percentage of women in selected publicly funded Research programs during 2019–2020. The percentage of submitted and granted projects led by women was around 30%. In 2021, approximately 50% of the 4377 members of the research groups integrated with CIBER (*Centro de Investigación Biomédica en Red*, https://www.ciberisciii.es/) in Spain were women, but only 26% of the group heads were women. Out of the 32 accredited Research Institutes of Health (*Institutos de Investigación Sanitaria*, IIS) in 2021, 22 had a medical doctor as a scientific director, with only 3 of them being physician women, accounting for 13.6% of the scientific director medical doctors (data not shown). The intensification program for research activity is a human resources initiative that allows attending physicians to dedicate time to lead their own research projects. Data show that the intensifications requested and granted to women were approximately 30% in 2019 and 2020, and 40% in 2021 (Table 3).

**Table 3:** Total number of medical doctors and percentage of women in selected publicly funded research programs, years 2019–2020. Source: Instituto de Salud Carlos III

	Submitted		Granted	
	Total *n*	Women *%*	Total *n*	Women *%*
Human Resources for Research	408	66.91	253	67.19
Allocation of partial waiver of health care activities for research (Intensification program)	137	35.04	109	29.36
Principal investigator in Health Research Projects	2246	36.46	842	32.54
Principal investigator in Biomedical Research Networks (CIBER)^a^	14	35.71	3	0.00
Principal investigator in Platforms for Research Support^b^	109	22.94	60	25.00

^a^Data from 2019 call only

^b^Data from 2020 call only

The WOMEDS results are also available at the following link of Tableau Public, a free platform to explore, create and publicly share data visualizations online [29]: https://public.tableau.com/app/profile/gender.medicine.

## Discussion

Limited evidence exists in the literature regarding gender inequity in medicine in Spain. To address this knowledge gap, the WOMEDS project was established. Its primary objective is to provide valuable insights into gender inequity in the field of medicine in Spain and contribute to ongoing efforts to promote gender equality. To achieve this, we plan to collect annual data to monitor the evolution of gender bias in leadership positions over time. The project focuses on presenting data and defining indicators related to various areas, including medical practice, representation of women in visible and influential roles within scientific and professional organizations, academia, and research. Whenever possible, the indicators were analyzed by medical specialty and by region. The data presented in this article cover the period from 2019 to 2021 and will be openly accessible through the project’s website at: https://womeds.es.

Our analysis of data from 2019 to 2021 confirms a significant gender inequity in leadership position within the medical field in Spain across all four settings examined. These findings align with previous studies that have consistently highlighted the lack of adequate representation and integration of women in top positions within the healthcare sector [2, 30]. Despite women gaining increased access to the medical profession, the disparity in leadership positions persists, underscoring the ongoing challenge of achieving gender equality in these areas.

A study focused on women’s leadership positions in various healthcare professions in Spain, including nursing, pharmacy, physiotherapy, medicine, dentistry, podiatry, and psychology revealed that only 16% of medical societies had a female president in 2014, compared to 77% nursing societies [21]. Among primary care societies, there was a higher proportion of women in executive positions (55%) compared to societies associated with hospital care specialties (28%)*.* A subsequent survey conducted five years later indicated that women were most represented in secondary leadership positions such as vice-secretary (or secretary in societies without vice-secretaries), member, vice-president, and vice-dean. The percentage of women in presidency positions or deanship was only 2% and 6%, respectively [31]. These findings highlight the ongoing need to continue efforts to achieve gender equity in leadership positions in Spain*.* Our study did not reveal a consistent pattern of feminization across specialties within the public health system. However, it is important to acknowledge that the data were only available from seven AACCs, which represents a limitation of this indicator. One specialty, preventive medicine, showed a clear advantage for female leadership, while a few others (neurosurgery, pneumology, and clinical neurophysiology) had WRs greater than or equal to 1 in three AACCs analyzed. It is crucial to recognize that gender does not correlate with intellectual competence, but women are often viewed less favorably than men, particularly in surgical disciplines. Our findings indicated that half of the specialties with low women empowerment in the health public system were from the surgical field, which is consistent with previous reports from other countries [32, 33]. This concern begins at very early stage, as 75% of medical students aspiring to pursue surgical careers reporting receiving verbal discouragement [34]*.* The probability of female doctors accessing the most demanded specialties for in Spain has been negatively influenced by changes introduced in the medical residency selection process in 2010. The main change was an increase in the importance placed on the results of the resident medical intern (MIR) test score, at the expense of the weight given to medical undergraduate studies. Specifically, the weight given to the MIR exam increased from 75 to 90%, while the contribution of the grade point average decreased from 25 to 10% [35].

The considerable variation in WRs of top positions in the public healthcare system across AACCs emphasizes areas where improvements can be made. It is possible to conduct a benchmarking exercise by examining the case of Navarra, where no evidence of gender bias in top positions was found. Our research provides valuable insights by identifying AACCs demonstrating better gender equity. Regional human resources managers in other regions can learn from their success and gain insights into the factors that contribute to it. Additionally, by comparing the four settings, our study can assist in establishing priorities for action among the different government ministries and agencies responsible for implementing measures.

Our study revealed a clear gender inequity in national medical congresses. Out of 36 Spanish societies that provided data from annual congresses held during the study period, only 4 medical societies had a WR greater than one for women speakers. Similarly, only 5 societies reported a WR greater than one for members in the scientific committee, indicating a significant imbalance against women. Additionally, throughout the entire period from 2019 to 2021, only 3 medical associations in Spain had a female president. These findings align with data published by international societies, such as the European Society of Medical Oncology, which also highlighted a significant underrepresentation of women as invited speakers at oncology congresses and as board members of oncology societies [36]*.*

Furthermore, we also identified a gender gap in the top leadership and institutional representation positions within provincial official colleges of physicians, academies of medicine, and universities. It is common to observe a man as president and a woman as vice-president or secretary in most medical organizations. These results are consistent with evidence from around the world [3, 8, 37–41] and within Spain [19–21, 23, 24], as documented in previous studies.

The situation in universities is worrying with a striking lack of women in leadership positions, according to other studies conducted in Spain as well as elsewhere [30, 42, 43]. The lack of women in top positions is striking, as evidenced by the fact that in 2020, only 9 out of 39 Spanish medical schools had women deans, compared to just two in 2010 [44]. These findings align with existing literature, which also highlights the presence of gender stereotypes in the highest-ranking decanal positions in US medical schools, with men primarily occupying clinical affairs and research affairs deanships, while women are more commonly found in admissions, diversity affairs, faculty affairs, and student affairs deanships [45]. Additionally, our research identified a gender gap in applications to research projects funded by the ISCIII, with fewer applications submitted by women. Moreover, women experienced lower success rates and received less average funding, which is consistent with evidence found globally [43, 46, 47]. These disparities in research funding could potentially contribute to the gender inequity observed in female first authorship manuscripts in scientific fields [13]. Based on these inequities observed in Spain, we propose the implementation of active policies of positive discrimination in calls for research projects in the medical field. This suggestion is in line with other authors who have recommended proactive efforts to promote gender equity in high-ranking work positions [48]. Recommendation for funders have also been put forward, such as describing the ideal candidate in non-gendered terms in grant proposals and reviewer guidelines, urging institutions to address possible gender inequities (e.g., salaries), and requesting recommenders to focus on an applicant’s objective research record rather than irrelevant personal circumstances [48].

Thanks to the #LancetWomen initiative, which called for papers addressing gender equity in science, medicine, and global health, important discussions have been initiated regarding the representation, experience, and promotion of women in these fields [49]. Various strategies have been proposed to promote gender diversity and inclusion in medicine, emphasizing the need for comprehensive interventions that address structural and systemic changes rather than focusing solely on individual attitudes and behaviors [50, 51]. These strategies include treating gender equality as an innovation challenge, changing institutional norms, fostering a culture of personal responsibility for change, implementing behavioral guidelines and action plans, and establishing organizational accountability. One key issue identified is the limited visibility of women in top leadership positions within medical organizations, which hinders the provision of role models for future generations [39]. A common pattern observed across various areas of analysis, including scientific societies, professional associations, and medical schools, is the predominance of men in visible head and institutional representative roles (e.g., president, dean), while women are often found in lower-ranking positions (e.g., vice-president, committee members). This bias is persistent and significant. An important objective of this study is to raise awareness of this problem and contribute its resolution. In fact, the completion of the questionnaires played a crucial role in making some societies and institutions aware of the gender gap for the first time.

The study has several strengths and limitations. Among its strengths, this is the first comprehensive study containing primary and recent data concerning various aspects of medical professions in Spain. This allowed us to report an exhaustive scenario of gender inequity in medicine in Spain. There are no similar studies published; most papers are focused on specific areas (for instance, women in the academy) or one specific specialty, but they did not cover all the angles (clinical practice, research, academia, and management) and different specialties at the same time.

However, there are certain limitations to consider. The study lacks historical data, preventing the analysis of the evolution of gender inequity over time. Nevertheless, the WOMEDS project aims to continue collecting annual data to monitor the evolution of gender bias in the medical profession in Spain. Another limitation is the absence of relevant information such as age and ethnicity, which are known to play significant roles in career advancement. Additionally, the intersectionality of gender with race, ethnicity, caste, or religion further exacerbates the disadvantage experienced in different parts of the world [3]. Another potential limitation is the bias associated with the sources of information, particularly with regard to medical specialties. Furthermore, the granularity and detail of the responses varied among medical societies. Currently, the research data only cover one program, but there are plans to expand the WOMEDS project with additional indicators and new collaborators who are willing to share their own data publicly for the analysis of gender bias and its evolution over time.

## Conclusion

Despite the predominance of female physicians in the public healthcare workforce across all regions in Spain, a significant underrepresentation of women persists in high-level leadership positions. Our study provides compelling evidence of pronounced gender inequity in Spain within the four settings analyzed. These findings highlight the urgent need for targeted policies and interventions at multiple levels (micro, meso, and macro) and within various institutions to address this issue.

### Supplementary Information

**Additional file 1: Table S1.** Total number of members, percentage of women members and percentage of women presidents according to Spanish medical societies, 2019-2021. **Figure S1.** Women Ratio in speakers invited in National Medical Congress according to Spanish medical societies, 2019–2021. **Figure S2.** Women Ratio in Members of the Scientific Committee in National Medical Congress according to Spanish medical societies, 2019–2021. **Figure S3.** Percentage of women on the board of directors by provinces, official colleges of physicians 2021. **Figure S4.** Percentage of women on the board of directors by regions, academies of medicine 2021. **Figure S5.** Percentage of Technological development projects in health admitted, granted, and succeeded, along with the average funding per project according to sex, 2020. **Figure S6.** Percentage of AC International Joint Programming admitted, granted, and succeeded, along with the average funding per project according to sex, 2020. **Figure S7.** Percentage of Independent clinical research projects admitted, granted, and succeeded, along with the average funding per project according to sex, 2020. **Figure S8.** Percentage of Health research projects admitted, granted, and succeeded, along with the average funding per project according to sex, 2020. **Figure S9.** Percentage of State Knowledge Generation Subprograms admitted, granted, and succeeded, along with the average funding per project according to sex, 2020.

## Data Availability

The datasets used and analyzed during the current study are available from the corresponding author on reasonable request.
