# Supplementary material for: Gender inequity in the medical profession: the women doctors in Spain (WOMEDS) study
Source: Hum Resour Health. 2023 Sep 20;21:77. doi: 10.1186/s12960-023-00860-2 (PMC10512601; doi:10.1186/s12960-023-00860-2)

**Additional file**

Manuscript title

GENDER INEQUITY IN THE MEDICAL PROFESSION:

THE WOMEN DOCTORS IN SPAIN (WOMEDS) STUDY

Table of Contents

[**Table S1**. Total number of members, percentage of women members and percentage of women presidents according to Spanish medical societies, 2019-2021. 2](#_Toc138678388)

[**Figure S1.** Women Ratio in speakers invited in National Medical Congress according to Spanish medical societies, 2019-2021. 3](#_Toc138678389)

[**Figure S2.** Women Ratio in Members of the Scientific Committee in National Medical Congress according to Spanish medical societies, 2019-2021. 4](#_Toc138678390)

[**Figure S3.** Percentage of women on the board of directors by provinces, official colleges of physicians 2021. 5](#_Toc138678391)

[**Figure S4.** Percentage of women on the board of directors by regions, academies of medicine 2021. 6](#_Toc138678392)

[**Figure S5.** Percentage of Technological development projects in health admitted, granted, and succeeded, along with the average funding per project according to sex, 2020. 7](#_Toc138678393)

[**Figure S6.** Percentage of AC International Joint Programming admitted, granted, and succeeded, along with the average funding per project according to sex, 2020. 8](#_Toc138678394)

[**Figure S7.** Percentage of Independent clinical research projects admitted, granted, and succeeded, along with the average funding per project according to sex, 2020. 9](#_Toc138678395)

[**Figure S8.** Percentage of Health research projects admitted, granted, and succeeded, along with the average funding per project according to sex, 2020. 10](#_Toc138678396)

[**Figure S9.** Percentage of State Knowledge Generation Subprograms admitted, granted, and succeeded, along with the average funding per project according to sex, 2020. 11](#_Toc138678397)

# **Table S1**. Total number of members, percentage of women members and percentage of women presidents according to Spanish medical societies, 2019-2021.

| **Abbreviation** | **Medical Society** | **Total members** | **% Women members** | **% Women president^a^** |
| --- | --- | --- | --- | --- |
| AEDV | Dermatology and venereology | 2856 | 43.3 | 0.0 |
| AEBM - ML | Biopathology and laboratory | 1337 | 57.1 | 0.0 |
| AEC | Surgery | 5408 | 42.3 | 0.0 |
| AEP | Pediatrics | 11,924 | 65.9 | 83.3 |
| AEU | Urology | 2654 | *na* | *na* |
| SEAIC | Allergology | 1595 | 64.1 | 0.0 |
| SEAP-IAP | Anatomical pathology | 1844 | 68.2 | 0.0 |
| SEDAR | Anesthesiology | 4122 | 54.4 | 0.0 |
| SEACV | Vascular surgery | 930 | 39.4 | 0.0 |
| SEQC-ML | Laboratory medicine | 2826 | 67.1 | 100.0 |
| SEC | Cardiology | 4761 | 35.9 | 0.0 |
| SECOMCyC | Oro-Maxillo-facial surgery | 906 | 31.6 | 0.0 |
| SECOT | Orthopaedics and traumatology | 6711 | 26.0 | 0.0 |
| SECPRE | Plastic, reconstructive and aesthetic surgery | 899 | 31.7 | 66.7 |
| SECT | Thoracic surgery | 491 | 31.0 | 0.0 |
| SEEN | Endocrinology | 1900 | *65.0* | 66.7 |
| SEFC | Clinical pharmacology | 348 | 54.3 | 100.0 |
| SEGG | Geriatrics and gerontology | *2547* | *61.1* | *0.0* |
| SEHH | Haematology and hemotherapy | 2807 | 65.5 | *0.0* |
| SEMFYC | Family and community medicine | 22,000 | 70.0 | 0.0 |
| SEMICYUC | Intensive care | 2757 | *56.3* | 33.3 |
| SEMI | Internal medicine | 7712 | 50.5 | 0.0 |
| SEMNIM | Nuclear medicine | 827 | 57.6 | 33.3 |
| SEMPSPH | Preventive medicine and public health | 367 | *na* | *na* |
| SEMG | General and family medicine | 7403 | 48.8 | 0.0 |
| S.E.N | Nephrology | 2561 | 57.2 | 100.0 |
| SEPAR | Pneumology | 4893 | 55.1 | 0.0 |
| SEN | Neurology | 1500 | 53.3 | 0.0 |
| SEO | Ophtalmology | 2739 | 43.5 | 0.0 |
| SEOM | Medical oncology | 2957 | 59.9 | 33.3 |
| SEOR | Radiation oncology | 1195 | 63.4 | 0.0 |
| SEORL-CCC | Otorhinolaryngology | 2921 | 39.8 | 0.0 |
| SEPD | Gastroenterology | 2424 | 54.2 | 0.0 |
| SEP | Psychiatry | 1357 | 48.4 | 0.0 |
| SERAM | Radiology | 7335 | 50.6 | 33.3 |
| SER | Rheumatology | 1976 | 55.7 | 0.0 |

^a^ The percentage of women presidents was calculated as the years covered by a woman president divided by the entire observed period, i.e. the 3-years 2019-2021.

# **Figure S1.** Women Ratio in speakers invited in National Medical Congress according to Spanish medical societies, 2019-2021.


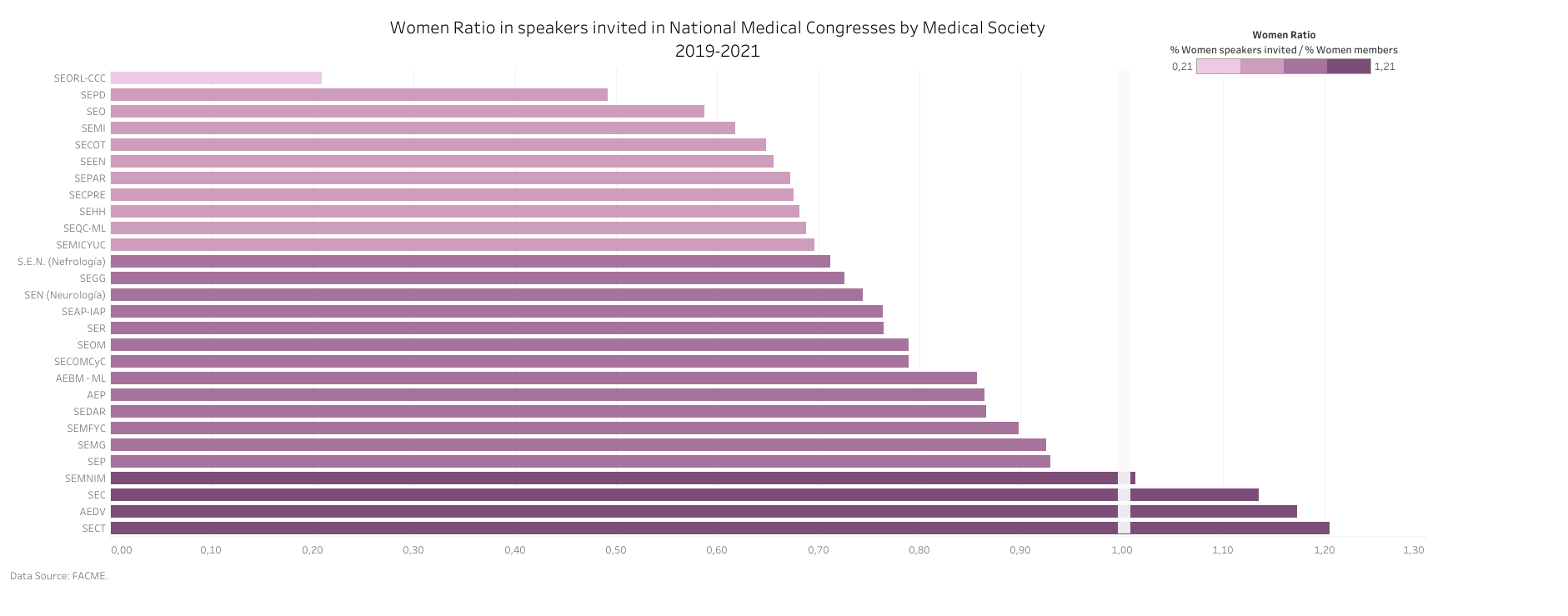


# **Figure S2.** Women Ratio in Members of the Scientific Committee in National Medical Congress according to Spanish medical societies, 2019-2021.
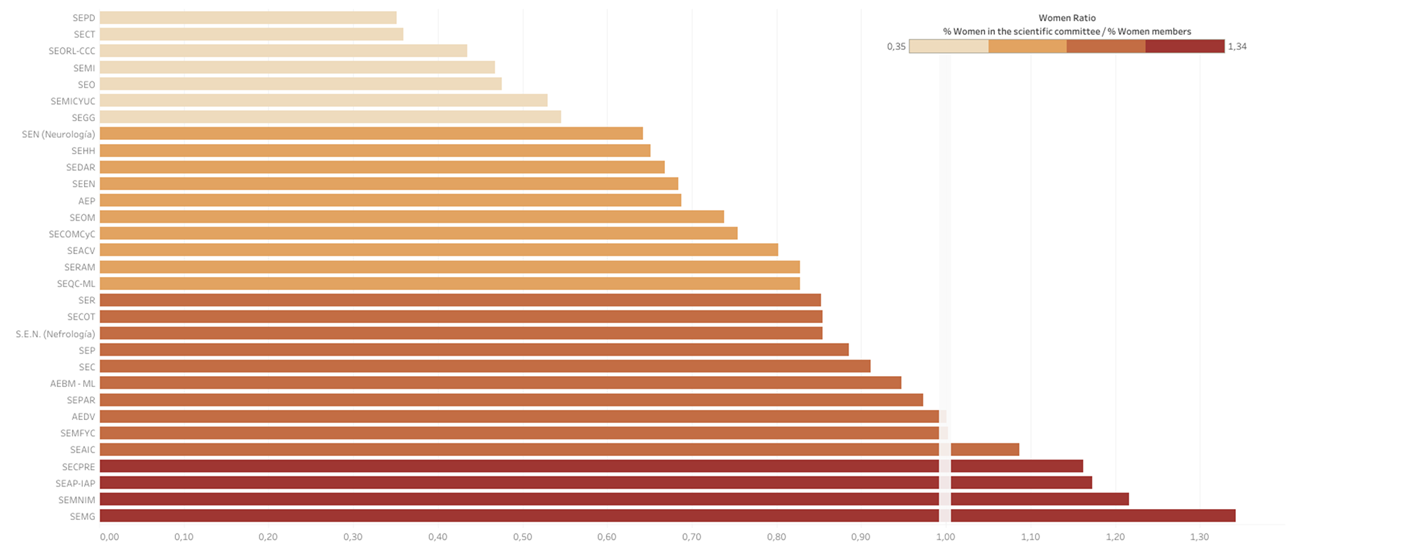


# **Figure S3.** Percentage of women on the board of directors by provinces, official colleges of physicians 2021.

**
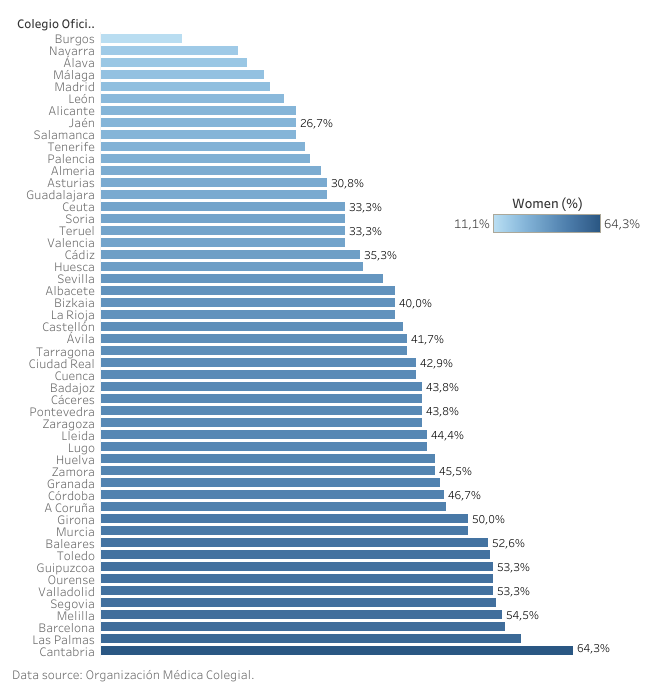
**

# **Figure S4.** Percentage of women on the board of directors by regions, academies of medicine 2021.

**
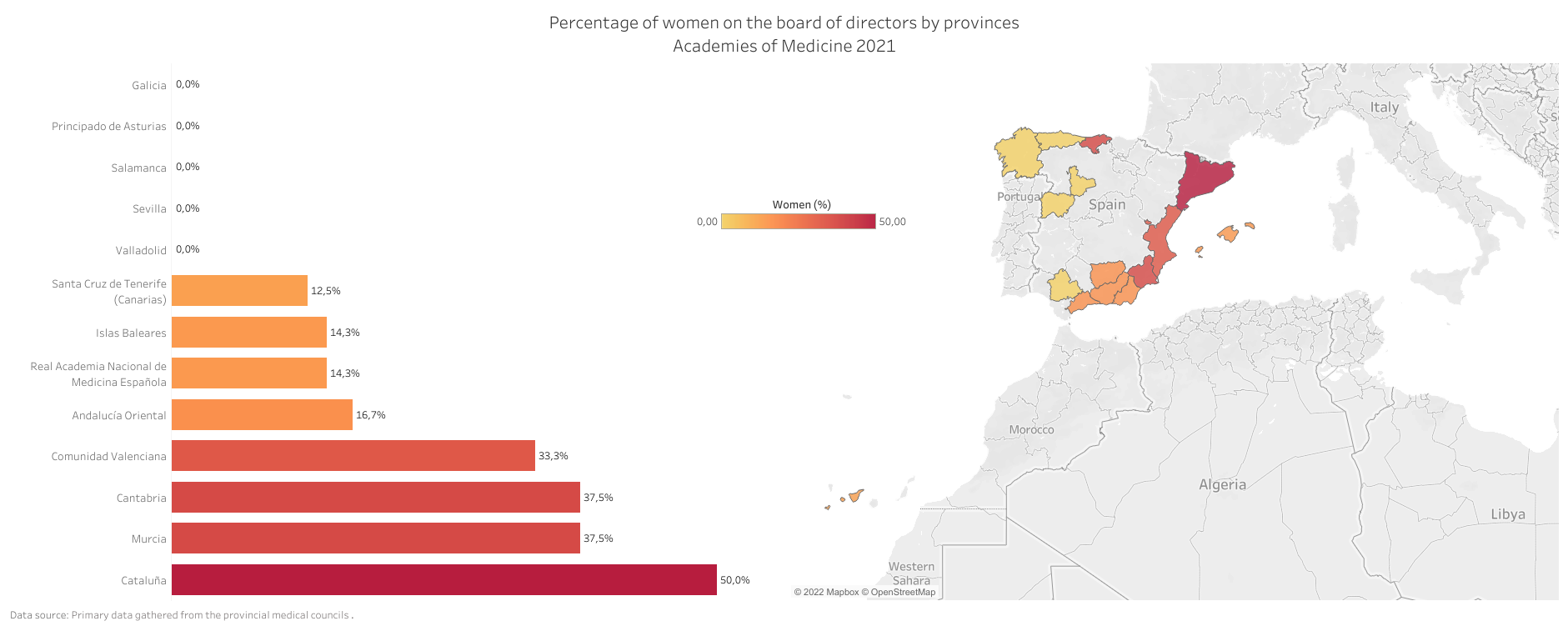
**

# **Figure S5.** Percentage of Technological development projects in health admitted, granted, and succeeded, along with the average funding per project according to sex, 2020.

**
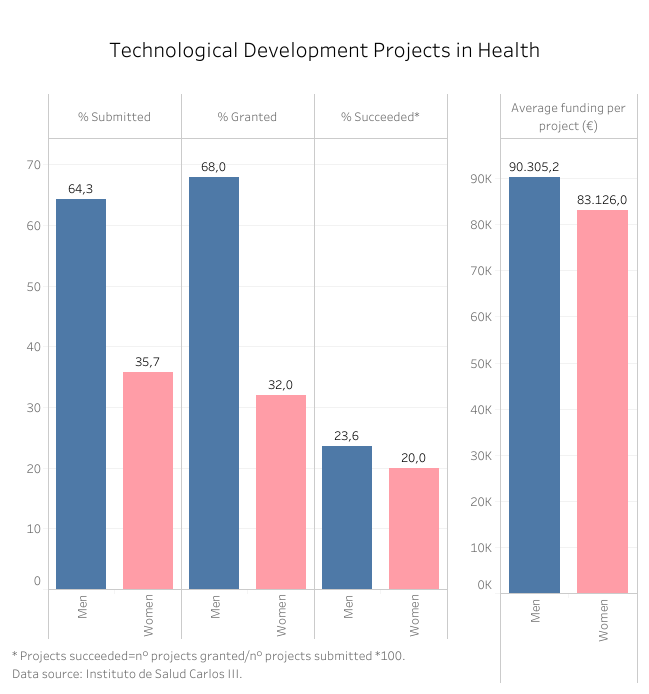
**

# **Figure S6.** Percentage of AC International Joint Programming admitted, granted, and succeeded, along with the average funding per project according to sex, 2020.

**
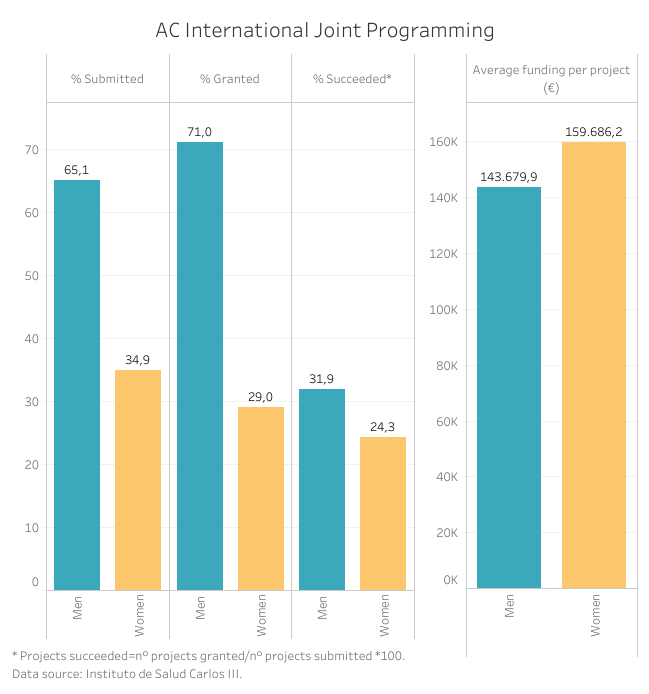
**

# **Figure S7.** Percentage of Independent clinical research projects admitted, granted, and succeeded, along with the average funding per project according to sex, 2020.

**
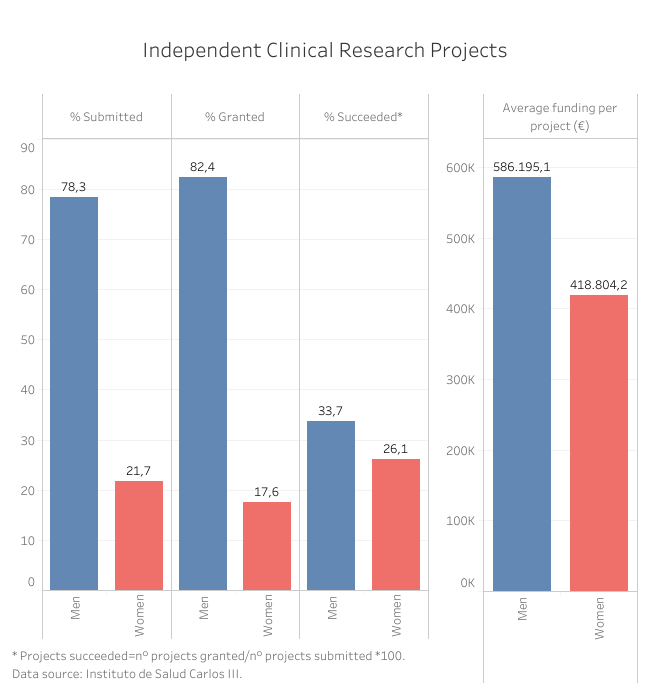
**

# **Figure S8.** Percentage of Health research projects admitted, granted, and succeeded, along with the average funding per project according to sex, 2020.


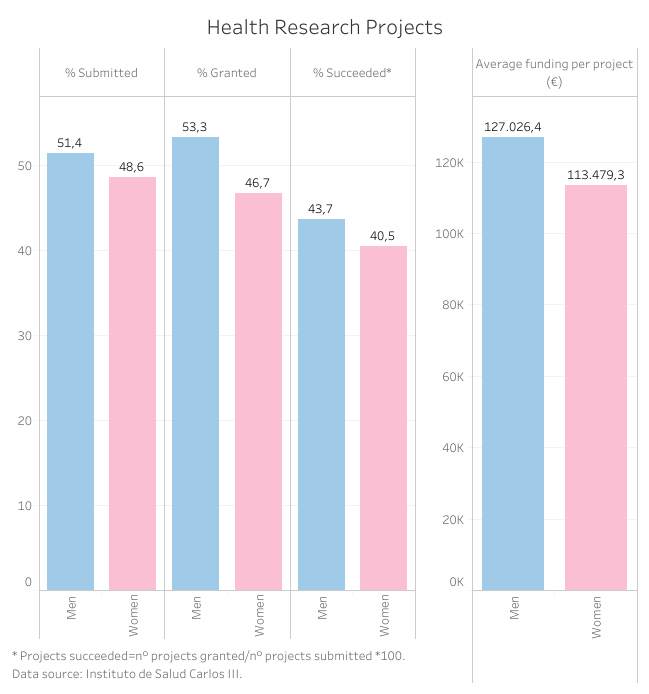


# **Figure S9.** Percentage of State Knowledge Generation Subprograms admitted, granted, and succeeded, along with the average funding per project according to sex, 2020.


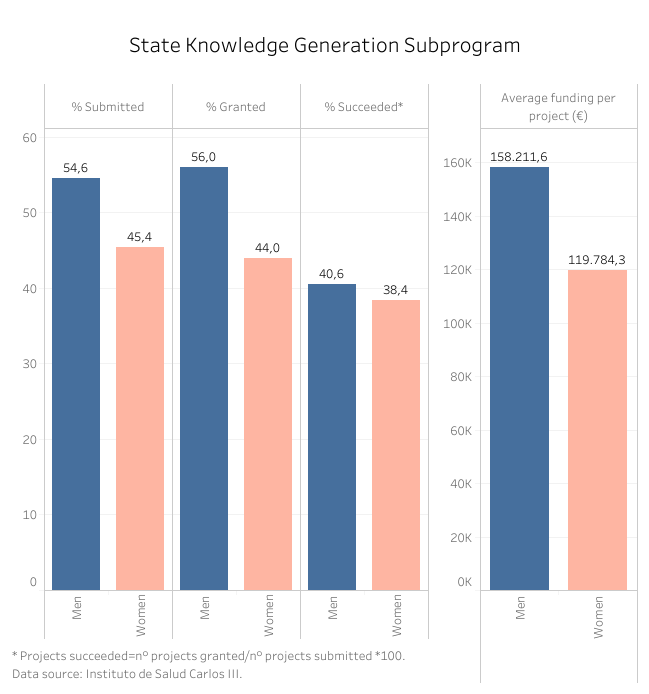

Supplement: Supplementary file 1 — Additional file 1: Table S1. Total number of members, percentage of women members and percentage of women presidents according to Spanish medical societies, 2019-2021. Figure S1. Women Ratio in speakers invited in National Medical Congress according to Spanish medical societies, 2019–2021. Figure S2. Women Ratio in Members of the Scientific Committee in National Medical Congress according to Spanish medical societies, 2019–2021. Figure S3. Percentage of women on the board of directors by provinces, official colleges of physicians 2021. Figure S4. Percentage of women on the board of directors by regions, academies of medicine 2021. Figure S5. Percentage of Technological development projects in health admitted, granted, and succeeded, along with the average funding per project according to sex, 2020. Figure S6. Percentage of AC International Joint Programming admitted, granted, and succeeded, along with the average funding per project according to sex, 2020. Figure S7. Percentage of Independent clinical research projects admitted, granted, and succeeded, along with the average funding per project according to sex, 2020. Figure S8. Percentage of Health research projects admitted, granted, and succeeded, along with the average funding per project according to sex, 2020. Figure S9. Percentage of State Knowledge Generation Subprograms admitted, granted, and succeeded, along with the average funding per project according to sex, 2020. [file 12960_2023_860_MOESM1_ESM.docx]
